# Deploying Fourier Coefficients to Unravel Soybean Canopy Diversity

**DOI:** 10.3389/fpls.2016.02066

**Published:** 2017-01-19

**Authors:** Talukder Z. Jubery, Johnathon Shook, Kyle Parmley, Jiaoping Zhang, Hsiang S. Naik, Race Higgins, Soumik Sarkar, Arti Singh, Asheesh K. Singh, Baskar Ganapathysubramanian

**Affiliations:** ^1^Department of Mechanical Engineering, Iowa State UniversityAmes, IA, USA; ^2^Department of Agronomy, Iowa State UniversityAmes, IA, USA; ^3^Department of Electrical and Computer Engineering, Iowa State UniversityAmes, IA, USA; ^4^Plant Sciences Institute, Iowa State UniversityAmes, IA, USA

**Keywords:** canopy outline, elliptic Fourier, diversity panel, image processing

## Abstract

Soybean canopy outline is an important trait used to understand light interception ability, canopy closure rates, row spacing response, which in turn affects crop growth and yield, and directly impacts weed species germination and emergence. In this manuscript, we utilize a methodology that constructs geometric measures of the soybean canopy outline from digital images of canopies, allowing visualization of the genetic diversity as well as a rigorous quantification of shape parameters. Our choice of data analysis approach is partially dictated by the need to efficiently store and analyze large datasets, especially in the context of planned high-throughput phenotyping experiments to capture time evolution of canopy outline which will produce very large datasets. Using the Elliptical Fourier Transformation (EFT) and Fourier Descriptors (EFD), canopy outlines of 446 soybean plant introduction (PI) lines from 25 different countries exhibiting a wide variety of maturity, seed weight, and stem termination were investigated in a field experiment planted as a randomized complete block design with up to four replications. Canopy outlines were extracted from digital images, and subsequently chain coded, and expanded into a shape spectrum by obtaining the Fourier coefficients/descriptors. These coefficients successfully reconstruct the canopy outline, and were used to measure traditional morphometric traits. Highest phenotypic diversity was observed for roundness, while solidity showed the lowest diversity across all countries. Some PI lines had extraordinary shape diversity in solidity. For interpretation and visualization of the complexity in shape, Principal Component Analysis (PCA) was performed on the EFD. PI lines were grouped in terms of origins, maturity index, seed weight, and stem termination index. No significant pattern or similarity was observed among the groups; although interestingly when genetic marker data was used for the PCA, patterns similar to canopy outline traits was observed for origins, and maturity indexes. These results indicate the usefulness of EFT method for reconstruction and study of canopy morphometric traits, and provides opportunities for data reduction of large images for ease in future use.

## Introduction

Soybean [*Glycine max* (L.) Merr.] is a leguminous plant and an important source of protein and oil for a wide range of end users across the world. Given the recent rate of climatic change and predictions for worsening environmental conditions, coupled with a growing global population (O'Neill et al., 2010), breeders are pressed to meet multi-objective requirements (increasing yield, decreasing resource requirement, increasing stress resiliency) during breeding. A promising approach to breeding involves rapid and accurate screening of a large number of plots under various conditions, in order to identify genotypes that maximize production potential under specific or broad environments and allow mapping the genes conditioning adaptation to varying stresses.

A critical aspect to this approach is accurate measurement of traits important to key physiological processes of the plant, which will enable breeders to evaluate and select from the pool of genetic diversity. It also follows that starting from a large, diverse pool of genetic diversity is important to efficiently attain these multi-objective requirements. It is imperative to note that the amount of genetic diversity used in modern soybean breeding is severely limited (Gizlice et al., 1994); therefore, the potential for finding useful alleles for traits of interest is very high by looking at the larger collection of soybean diversity. However, to characterize the genetic diversity present in germplasm, methods that are amenable to high throughput as well as automated applications are required. This is because quantifying traits manually/visually is difficult (especially when considering large, diverse planting populations), and is often done with high error or user bias. High throughout phenotyping along with computer vision has enabled a revolution in crop phenotype collection to reduce reliance on visual ratings while improving accuracy (Singh et al., 2016). Image based phenotyping allows the collection of plant and canopy morphological traits on spatial and temporal scales enabling the monitoring of physiological development and to quantify abiotic and biotic stresses (Pauli et al., 2016).

In this context, our focus is on evaluating the soybean canopy outline. Yield potential is a function of products of incident solar radiation, conversion, and partitioning efficiencies, and linear improvements have been observed in each of the three efficiencies (Koester et al., 2014). For continued yield increase these three efficiencies need to be increased, therefore, necessitating continued research on canopy traits. Canopy outline is important to evaluate light interception ability. Light interception, measured as a function of ground surface area shaded by at least one leaf, directly factors in to the yield potential equation (Koester et al., 2014). Canopy closure rates will affect the light interception rate of soybean by capturing incident sunlight sooner over a greater area. Increased canopy closure rates also have secondary effects by inhibiting weed species germination and emergence, which may provide a source of protection against difficult to control weeds (Harder et al., 2007; Evers and Bastiaans, 2016). We therefore, focus on traits that quantify soybean canopy outline and structure that are important to evaluate differences in light interception ability, canopy closure rates, and row spacing response. To analyze the canopy outline, generally, digital image of a canopy is captured by high resolution cameras. Traditional morphometric traits including aspect ratio, roundness, circularity, and solidity (Olson, 2011; Chitwood et al., 2014a), while useful are not sufficient to capture the complexity of shapes, especially when considering shape evolution. In this manuscript, we utilize a methodology [based on Elliptic Fourier descriptors (EFD)] that provides reduction in data, enables visualization of diversity and results in a rigorous quantification of shape. The EFD has been used in various studies in past for plant species identification (Neto et al., 2006), tomato leaf shape (Chitwood et al., 2014a), leaf shape and venation (Chitwood et al., 2014b), flower petal shape (Iwata and Ukai, 2002; Yoshioka et al., 2004) and soybean pod diversity (Truong et al., 2005). In the present study EFD is used for soybean canopy outline diversity analysis.

We collected canopy images in a replicated field experiment using 446 soybean PI lines acquired from the USDA soybean germplasm collection. This is a very diverse set of germplasm, from over 25 different countries exhibiting a wide variety of maturity, seed weight, and stem termination. Digital images of the canopy were available for analysis, which were taken using a standard imaging protocol (Zhang et al., submitted). The images produced 8 GB of data. We deployed the analysis framework to reduce the useful storage size required while retaining information related with canopy outline, and evaluate shape descriptors/traits to investigate shape diversity among the lines. This paper represents the first stage of our analysis program. After evaluating and quantifying the shape diversity exhibited by this set of lines, we envision (in subsequent work) utilizing this framework to analyze and evaluate time-dependent geometric canopy traits to isolate genetic pathways that control various stress adaptation mechanisms.

## Materials and methods

### Plant material, field phenotyping and data acquisition

**Figure 1:**
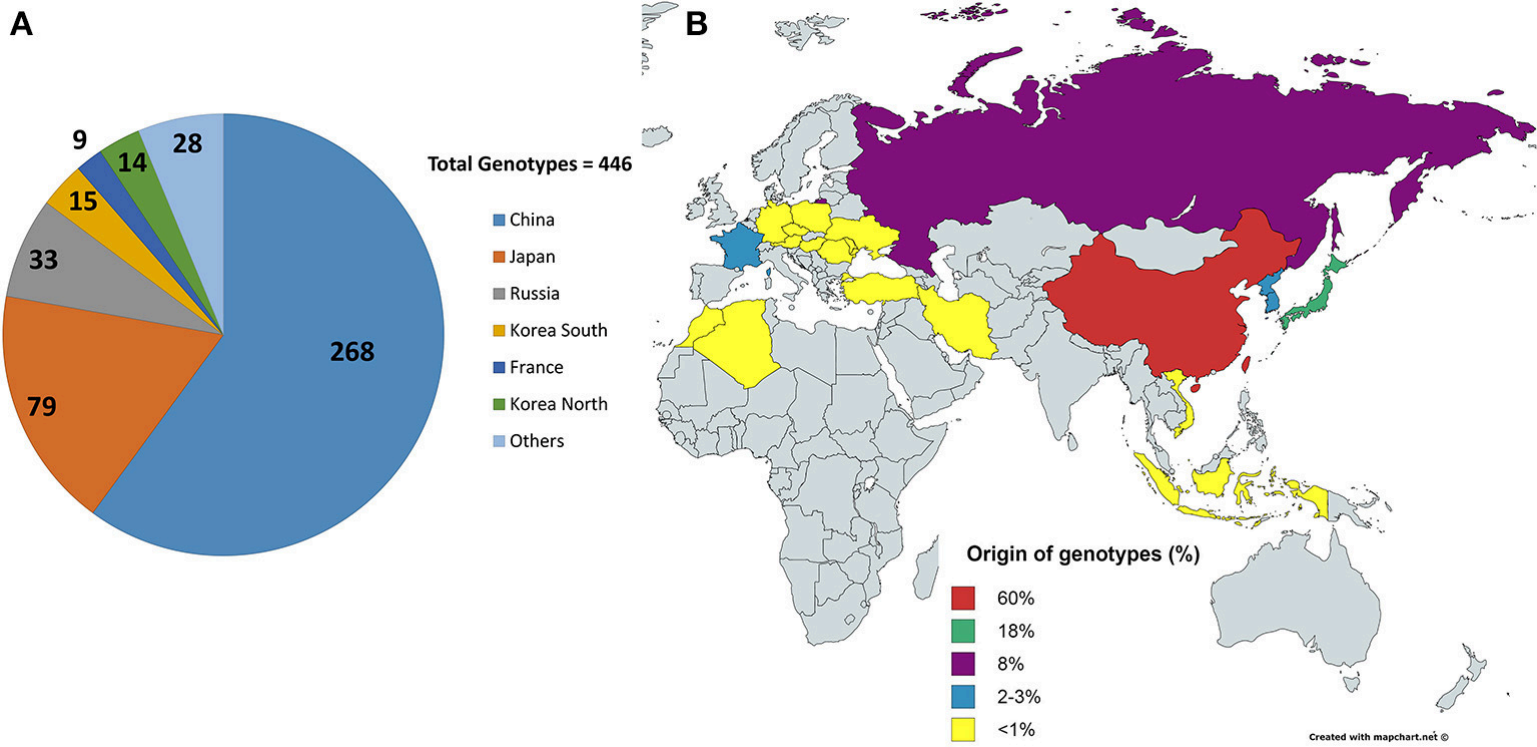
**Origins of the lines. (A)** Shows the distribution of the 446 lines/genotypes based on origins, collected from USDA soybean line collection. China and Japan are the highest contributors in the selected collection. **(B)** Shows the origins in terms of percentage contribution from different countries. Yellow shows countries with <1% contribution, i.e., four genotypes or less.

A total of 446 soybean PI lines from 25 different countries acquired from the USDA soybean germplasm collection, grown near Ames, IA, in 2015, were used for this study (Figure 1). These lines also differ from one another in terms of maturity, seed weight, and stem termination. The field experiment was organized as a randomized complete block design with four replications. The germplasm accessions were planted in hill plots of five seeds per plot at one foot between plots and two feet between rows. The images were taken at 2 weeks after second trifoliate leaf stage using a Canon EOS REBEL T5i camera with the Scene Intelligent Auto model. All images were stored in RAW image quality. An umbrella was always used to shade the area under the camera view, and the flash function was kept off to maintain consistent illumination. Also, at the beginning of imaging operations and every 20 min thereafter or whenever the light condition changed (e.g., cloud cover), a picture of the X-Rite Color Checker Color Rendition Chart was taken to calibrate the illumination and the color of canopy image. Whenever possible, weeds and other plant residuals, connected to the plant canopy in the view of camera, were removed for easy image processing and enhanced efficiency of subsequent image processing. After checking for quality of image (removing images that did not follow the imaging protocol, that exhibited disease symptoms or that did not have an intact canopy), around 1200 images were used for subsequent analysis. The single nucleotide polymorphism (SNP) dataset for the panel was prepared in a previous study (Song et al., 2013) and was acquired from the SoyBase site (http://soybase.org/).

### Image data processing

*First, each image was segmented*. Then they were converted from native RGB to hue-saturation-value format to efficiently segment the foreground (the plant) from the background. The background of an image (soil) contains more gray pixels than the foreground (plant) and lacks in green and yellow hue values; therefore, most of the background was removed by excluding pixels that had a saturation value below a predefined threshold and hue values outside of a predefined range. The saturation threshold value was obtained by identifying the saturation values of the background in 148 diverse images. The hue range was simply obtained from the hue color wheel, removing pixels that were neither green nor brown.

Once segmentation was done, the connected components labeling method (Suzuki et al., 2003; Gonzales and Woods, 2008; Wodo et al., 2012; Samudrala et al., 2013; Pace et al., 2014) was used on the processed image to remove spurious outliers and noise from the image (e.g., plant debris on soil). This was accomplished by identifying clusters of pixels that connected to one another, followed by labeling them, and identifying the largest connected component (i.e., plants in a plot). Cleaning was done by removing any other connected components that contained fewer pixels than the largest connected component. Then, a mask of the isolated plant was applied to the binary image. In contrast to other commonly used thresholding methods (Otsu, 1975; Browning and Browning, 2009; Lee et al., 2016; Naik et al., submitted), no significant pixel loss was observed.

**Figure 2:**
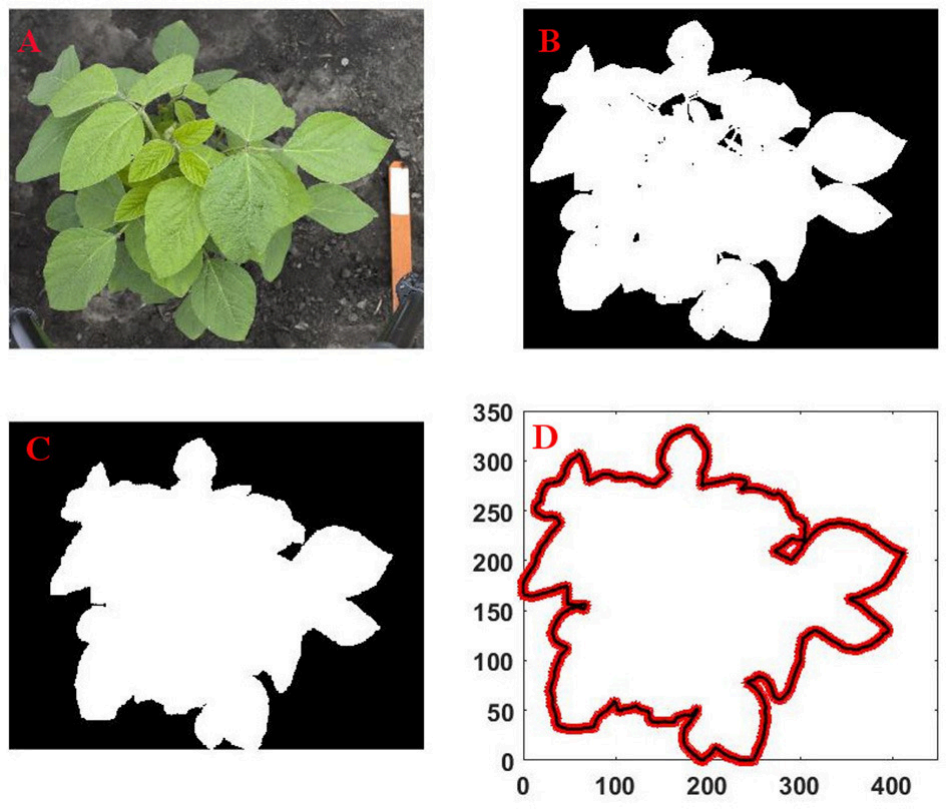
**Sequence of the canopy outline extraction process from an image captured by digital camera: (A)** original image; **(B)** cleanup, segmentation and conversation of the RGB image to binary image; **(C)** filling of empty spaces inside the binary image; **(D)** extraction of outline from the filled image.

*Next, contour/outline of canopy was defined from the locations of boundary pixels of the image*. Any empty space inside the canopy was filled, and the outline of the canopy was obtained from the filled binary image. The extracted outline of the canopy was represented as a sequence of x and y coordinates of boundary pixels (see Figure 2 for a schematic of the operation). The MATLAB image processing toolbox was for all image data processing operations.

### Geometric and shape descriptors details

Both traditional and EFD are utilized for subsequent analysis. The traditional shape descriptors/traits, including aspect ratio, roundness, circularity, and solidity are defined as follows:

- aspect ratio = the ratio of the major to minor axis of the best-fitted ellipse on the canopy;
- roundness = $4\frac{Area}{\pi {\left(Major\;axis\right)}^{2}}$; it indicates closeness of the shape of the canopy to a circle;
- circularity = $4\pi \frac{Area}{{\left(Perimeter\right)}^{2}}$; it indicates closeness of the form of the canopy to a circle;
- solidity = $\frac{Area}{Convex\;Area}$.

For EFD, the extracted outline was chain coded and expanded into a shape spectrum by obtaining the Fourier coefficients. Summations of the harmonics of the resulting series approximate the outline of the original shape. Each harmonic was represented by four set of Fourier coefficients. The coefficients are descriptors of the shape of the canopy outline/contour. Finally, the descriptors were made invariant of shape, rotation and starting-point of the contour by standardization (Kuhl and Giardina, 1982).

Each contour was chain coded following standard practice (Freeman, 1974). Then linear interpolation was used to represent contour between the two chain-coded points [e.g., (*i*−1)^th^ and *i*^th^]. The x and y coordinates of any point, *p*^th^, were expressed as

(1) $$\begin{array}{r} x_{p}=\overset{p}{\underset{i=1}{\sum }}\Delta x_{i}\text{ and }y_{p}=\overset{p}{\underset{i=1}{\sum }}\Delta y_{i} \end{array}$$

where Δ*x*_*i*_ and Δ*y*_*i*_ are the differences along the x and y axes between the (*i*−1)^th^ and the *i*^th^ points.

The length of the contour from the starting point to the *p*^th^ point and the perimeter of the contour are denoted by *t*_*p*_ and *T*, respectively.

(2) $$\begin{array}{r} t_{p}=\overset{p}{\underset{i=1}{\sum }}\Delta t_{i}\text{ and }T=t_{s} \end{array}$$

where Δ*t*_*i*_ is the distance between *i*−1^th^ and *i*^th^ points, and s is the total number of the chain coded points on the contour.

The co-ordinates in Equation (1) were expressed using elliptic Fourier expansion as

(3) $$\begin{array}{r} x_{p}=A_{0}+\overset{\infty }{\underset{N=1}{\sum }}\left(a_{N}\cos\frac{2N\pi t_{p}}{T}+b_{N}\sin\frac{2N\pi t_{p}}{T}\right) \end{array}$$

and

(4) $$\begin{array}{r} y_{p}=C_{0}+\overset{\infty }{\underset{N=1}{\sum }}\left(c_{N}\cos\frac{2N\pi t_{p}}{T}+d_{N}\sin\frac{2N\pi t_{p}}{T}\right) \end{array}$$

where the coefficients of the *N*^th^ harmonic were

(5) $$\begin{array}{r} a_{N}=\frac{T}{2N^{2}\pi^{2}}\overset{k}{\underset{p=1}{\sum }}\frac{\Delta x_{p}}{\Delta t_{p}}\left(\cos\frac{2N\pi t_{p}}{T}-\cos\frac{2N\pi t_{p-1}}{T}\right) \end{array}$$

(6) $$\begin{array}{r} b_{N}=\frac{T}{2N^{2}\pi^{2}}\overset{k}{\underset{p=1}{\sum }}\frac{\Delta x_{p}}{\Delta t_{p}}\left(\sin\frac{2N\pi t_{p}}{T}-\sin\frac{2N\pi t_{p-1}}{T}\right) \end{array}$$

(7) $$\begin{array}{r} c_{N}=\frac{T}{2N^{2}\pi^{2}}\overset{k}{\underset{p=1}{\sum }}\frac{\Delta y_{p}}{\Delta t_{p}}\left(\cos\frac{2N\pi t_{p}}{T}-\cos\frac{2N\pi t_{p-1}}{T}\right) \end{array}$$

(8) $$\begin{array}{r} d_{N}=\frac{T}{2N^{2}\pi^{2}}\overset{k}{\underset{p=1}{\sum }}\frac{\Delta y_{p}}{\Delta t_{p}}\left(\sin\frac{2N\pi t_{p}}{T}-\sin\frac{2N\pi t_{p-1}}{T}\right) \end{array}$$

For a shape representation using *N* harmonics, 4*N* coefficients were evaluated and were subsequently used as descriptors of the shape. The original shape of canopy outline was reconstructed using inverse Fourier transform from these shape descriptors. The accuracy of the reconstruction depends on the number of harmonics (*N*) used. We defined the deviation of the reconstructed shape from the original as

(9) $$\begin{array}{r} E_{N}=\frac{\text{max }\left[\sqrt{{\left(x_{p}-x_{N_{p}}\right)}^{2}+{\left(y_{p}-y_{N_{p}}\right)}^{2}}\right]}{\text{2L}}\;\times \;100\% \end{array}$$

where *x*_*N*_*p*__ and *y*_*N*_*p*__ are the approximated co-ordinates using *N* harmonics, and L is half of the length of the major axis (semi-major axis) of the best-fitted ellipse to the shape (estimation of L is presented in the standardized shape descriptors section). As with any approximation scheme, increase in the number of harmonics results in high accuracy of reconstruction, but requires more processing time and storage. We next detail an approach of identification of the optimal number of harmonics to use that represents a balance between accuracy and computational requirement.

### Choice of number of harmonic descriptors

**Figure 3:**
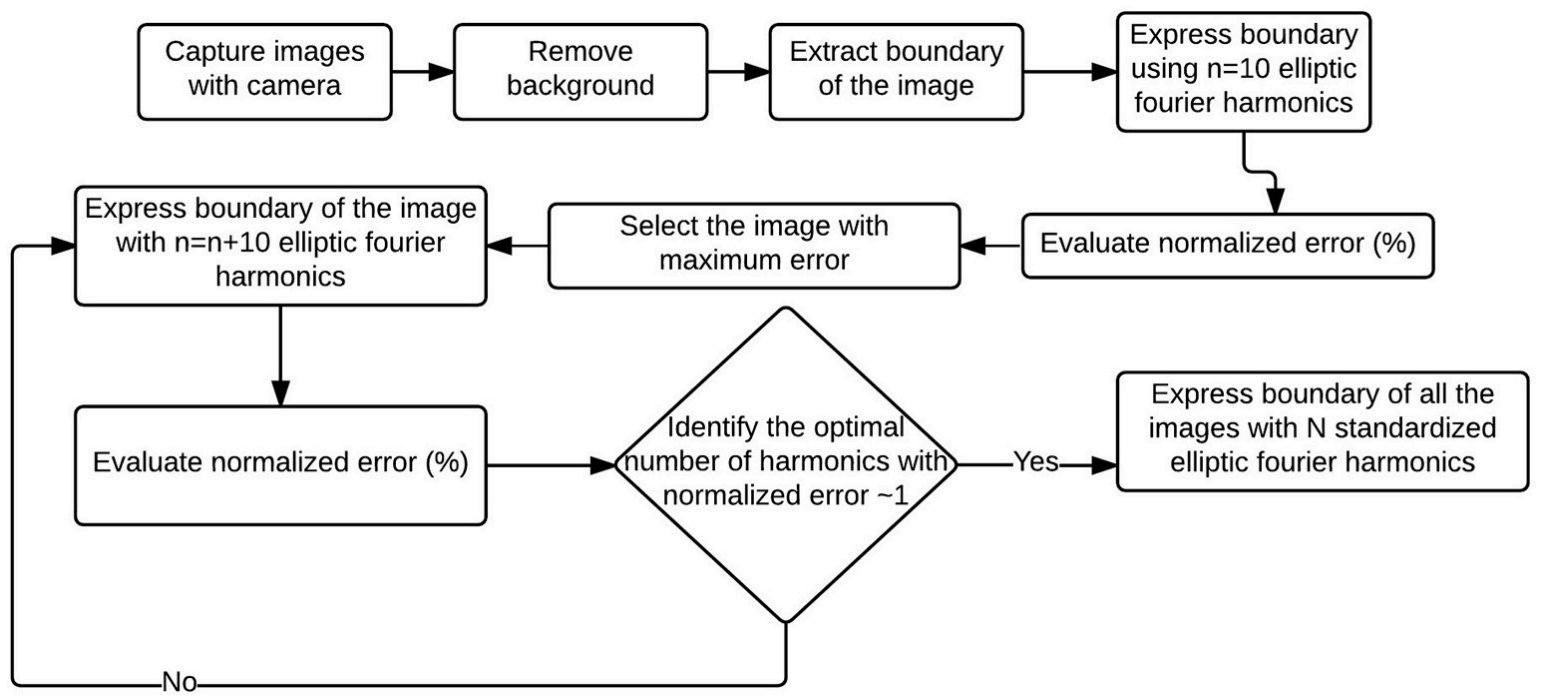
**Flowchart to evaluate EFDs for a set of images**. First canopy outline/boundary from all the images were captured and expressed using 10 elliptic Fourier harmonics. The worst (in terms of deviation from the original outline) reconstructed outline (which is also the most complex outline) from the EFDs was identified. Optimal number of harmonics of the complex outline was identified, and all the outlines were expressed using that optimal number of harmonics.

We first identified the most complex canopy (that would require the most number of harmonics to accurately represent) from the set of all available canopies. We then evaluated the optimal number of harmonics, *N*, needed to represent this canopy and subsequently utilize this number *N* for all other canopy descriptors. This ensures consistency while ensuring that a desired threshold of accuracy is met. Figure 3 illustrates this idea using a flowchart format.

**Figure 4:**
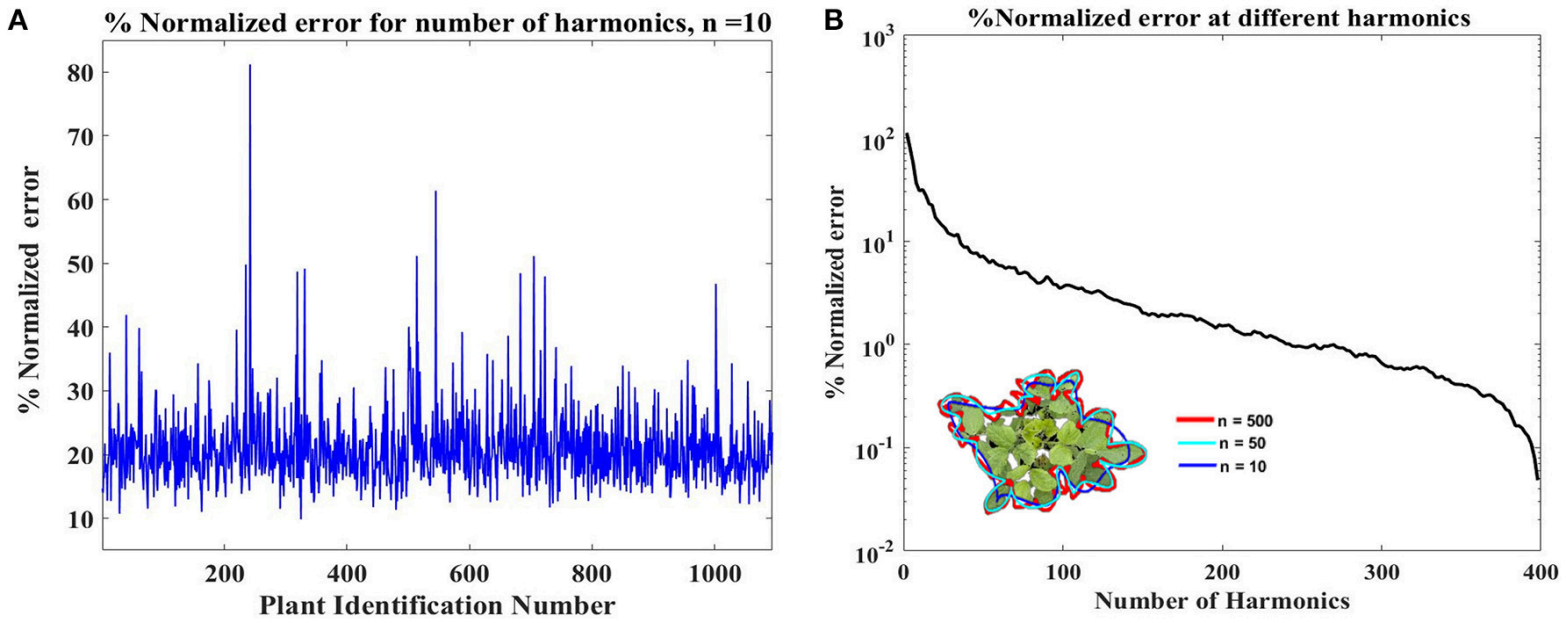
**(A)** Identification of the complex canopy outline in the dataset. The identification was performed by evaluating deviation of the reconstructed outlines using ten harmonics from the original outline (Equation 9). Plant with Id# 246 shows the highest deviation/error and was considered as the most complex canopy outline. **(B)** Identification of optimal harmonics for the plant Id# 246, shown in inset. The % normalized error value reaches near 1% around 500 harmonics, and the inset shows that 500 harmonics (red line) outlines is in good agreement with the original outline. This value, 500, was considered as the optimal number of harmonics.

To identify the most complex canopy, error between outline from each original image and the Fourier approximation using the first ten harmonics of that image was calculated using Equation 9. The canopy with the highest deviation exhibits the most complex shape (and is shown in Figure 4A). Harmonic representation of this canopy outline using increasing number of harmonics (from 10 to 1000 in steps of 10) was constructed and the error was computed. Figure 4B shows how the error decreases as the number of harmonics used for shape representation is increased. We chose *N* = 500 as this is where the normalized error reaches around 1%. Thus, each canopy outline is subsequently represented using a 500 harmonic representation.

### Standardized shape descriptors

The descriptors were made invariant in size, rotation, shift by standardizing the coefficients (Kuhl and Giardina, 1982), using the size and spatial location on the ellipse represented by the first harmonic. The standardized coefficients are

(10) $$\begin{array}{rcl} \left[\begin{array}{cc} a_{N}^{*} & b_{N}^{*}\\ c_{N}^{*} & d_{N}^{*} \end{array}\right] & = & \frac{1}{L}\left[\begin{array}{cc} \cos\psi & \sin\psi \\ -\sin\psi & \cos\psi \end{array}\right]\\ \left[\begin{array}{cc} a_{N} & b_{N}\\ c_{N} & d_{N} \end{array}\right]\left[\begin{array}{cc} \cos N\theta & \sin N\theta \\ -\sin N\theta & \cos N\theta \end{array}\right] \end{array}$$

where $L=\sqrt{\left[{\left(A_{0}-x_{m}\right)}^{2}+{\left(C_{0}-y_{m}\right)}^{2}\right]}$ is half of the length of the major axis of the ellipse from 1st harmonic, (*A*_0_, *C*_0_) is the center of the ellipse, (*x*_*m*_, *y*_*m*_) is the location of modified starting point (point on the major axis of the ellipse), $\theta =\frac{2\pi t_{m}}{T}$ and $\psi =\tan^{-1}\left[\frac{y_{m}-C_{0}}{x_{m}-A_{0}}\right]$, is the angle between the major axis of the ellipse and x axis. After standardization, three Fourier coefficients became constant ($a_{1}^{*}$ = 1, $b_{1}^{*}$ = 0 and $c_{1}^{*}$ = 0). An in-house code was developed (using MATLAB) to implement the above methods. This code is available upon request.

### Analysis procedure

After each canopy outline is represented using 500 Fourier shape descriptors, traditional morphometric traits are evaluated from the shape. For comparative assessment of the utility of Fourier shape descriptors, the traits obtained from EFD reconstructed canopy outlines are compared with traits obtained from the original images. Diversity of the traits among the lines/genotypes based on the country of origin is investigated. For visualization and interpretation of diversity, PCA on the EFD is next deployed. Data visualization based on the first two principle components is performed with a focus on four classifications including country of origin, maturity index, stem termination index and seed weight. Finally, the canopy descriptors that are suitable for genetic improvement were suggested.

### Trait heritability estimate

The model for the phenotypic trait with a single trial of randomized complete block design was *y*_*ik*_ = μ + *g*_*i*_ + *b*_*k*_ + *e*_*ik*_, where *y*_*ik*_ is the trait observation/estimate of the *i*^th^ genotype at the *k*^th^ block, μ is the total mean, *g*_*i*_ is the genetic effect of the *i*^th^ genotype, *b*_*k*_ is the block effect, and *e*_*ik*_, is a random error following N(0, σ^2^_e_). Accordingly, the broad-sense heritability on an entry-mean basis of each trait was calculated as H^2^ = $\sigma_{\text{g}}^{2}$/[$\sigma_{\text{g}}^{2}$ + $\sigma_{\text{e}}^{2}$/*k*], where $\sigma_{\text{g}}^{2}$ is the genotypic variance, *k* is the number of replications. The analysis of variance was implemented in R via the ANOVA function. The variance components were calculated with all effects considered to be random.

## Results and discussion

### Traditional morphometric traits evaluation and their fidelity

**Figure 5:**
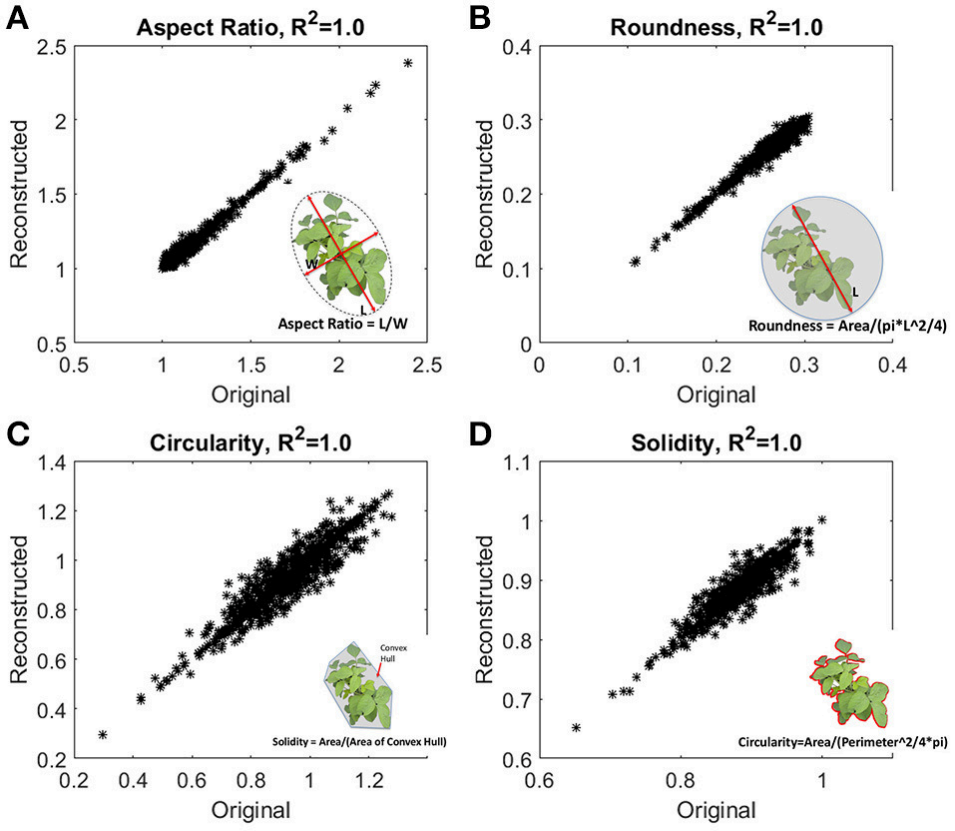
**Fidelity checks of the reconstructed outlines**. Traditional morphometric traits, **(A)** aspect ratio, **(B)** roundness, **(C)** circularity, and **(D)** solidity were evaluated from the reconstructed outlines, and also from the original images. Inset shows the definition of the traits. Aspect ratio and circularity are related with the shape of the outline, and roundness and solidity are related with the form or area enclosed by the outline.

The accuracy of reconstruction was checked by comparing traits obtained from original images and traits evaluated from the reconstructed outlines using 500 harmonics. Figure 5 shows excellent agreement with the traits evaluated from original images and reconstructed outlines. In our study, data size of an image captured by camera is ~15 MB (RAW format), and size of the 500 harmonics used for reconstruction is ~5 kB. In other words, EFD requires *two orders of magnitud*e less memory with minimal computational overhead to evaluate traditional traits.

### Shape diversity based on the traditional traits

**Figure 6:**
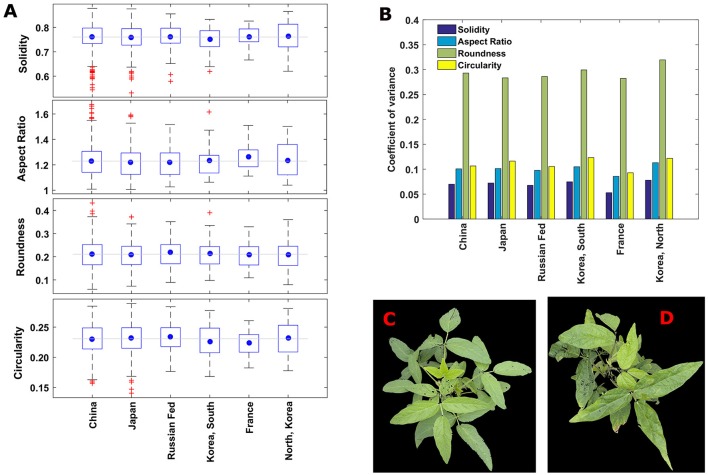
**(A)** Boxplots showing the variation of traditional traits among the plants with different countries of origin. The blue circle shows the mean value of a trait in an origin and gray horizontal line indicates overall mean value the trait including all the origins. **(B)** Coefficient of variance of the canopy morphometric traits among major geographical countries of origin. Roundness has the highest diversity and solidity has the lowest diversity in all the countries. **(C,D)** Representative images of canopies those are outliers in solidity (in **A**). **(C)** A canopy of line/genotype PI567159A from china and **(D)** a canopy of line/genotype PI594170B from Japan.

To investigate the shape diversity based on the traditional traits, the traits are grouped based the origin of the lines, and presented in box plots (Figure 6A). The plots indicate that lines from France have less spread in all the traits. Lines from North Korea have the highest spread in aspect ratio and circularity. In brief, there is considerable variation in traits regardless of country of origin. Roundness has almost three times more spread than the other traits for all countries (Figure 6B). Fifty-five individual plants were identified as canopy outline outliers (red “+” symbol in Figure 6A). Fifty lines were outliers in only one of the four replications, suggesting that non-genetic factors including differences in growth stage at imaging (such as due to delayed emergence) as well as unequal number of seeds per plot could be the causal factors for the observed variation. These fifty plants are removed from the data set for subsequent analysis.

Two lines are outliers for solidity, and occur as outliers in two of three reps (Figures 6C,D), i.e., around 67%. Six canopies were outliers for two traits simultaneously. These traits were paired as circularity with either solidity or aspect ratio, with two thirds of the circularity outliers also showing up as outliers for another trait. In spite of this, when comparing trait relationships using the complete data set, the pairings of (1) roundness with solidity, and (2) aspect ratio with circularity were the only two combinations with an |r| (correlation coefficient) >0.75. The genotypes lines with these outliers are strong candidates for extreme variation among canopy traits, and are interesting for additional studies to determine if they may provide unique ideotypes for practical applications. Furthermore, the study of genetic variation in canopy traits on a temporal and spatial scale will require inclusion of diverse germplasm in environments that may differ in latitude, longitude, water and nutrient stresses, planting date, and row spacing, in order to ultimately associate canopy traits with productivity and resiliency.

### Shape diversity based on EFDs

**Figure 7:**
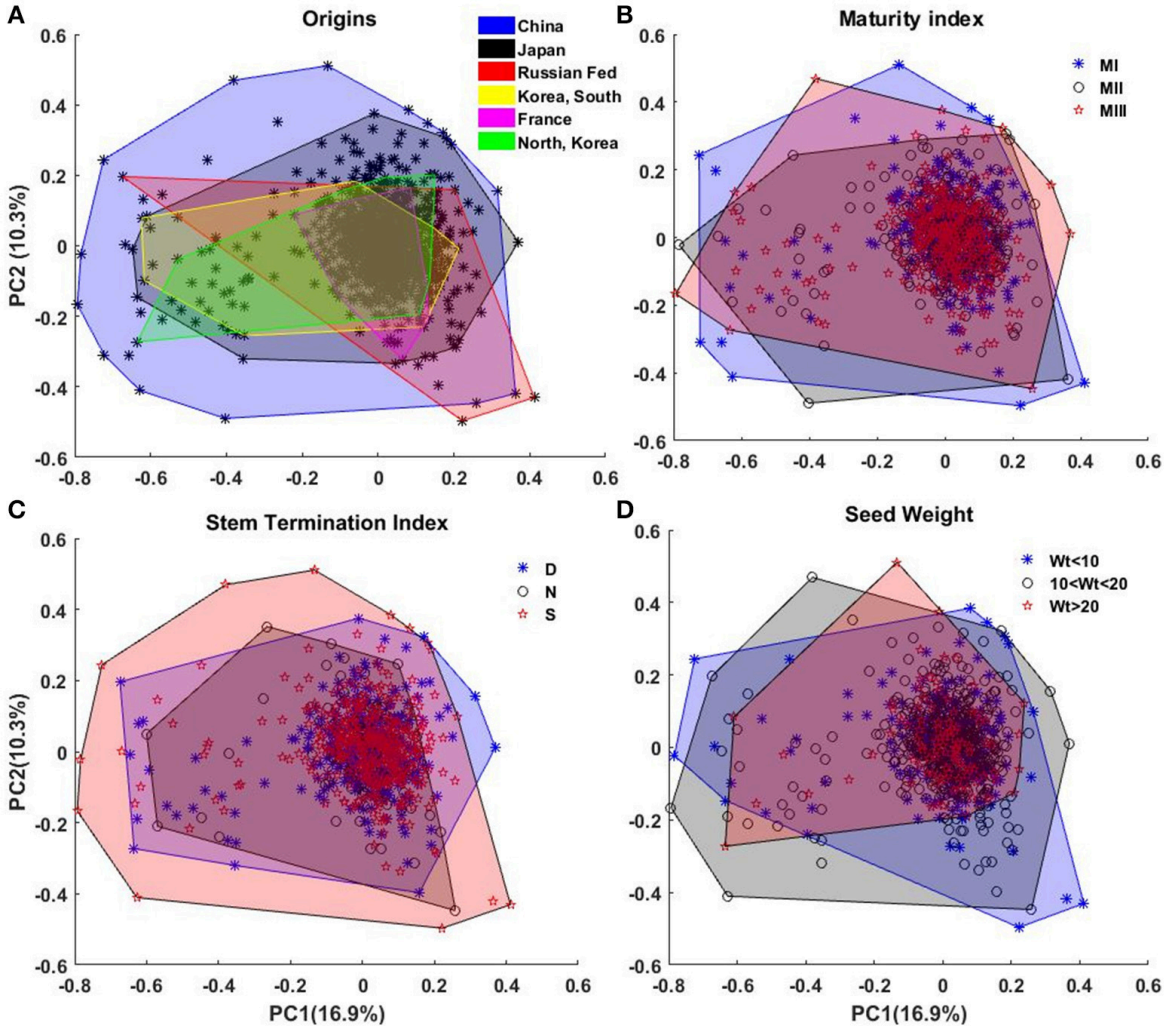
**Representation of the diversity of canopy outline among (A)** the origins, **(B)** maturity index, **(C)** stem termination index, and **(D)** weight of 100 seeds (in grams). In **(A)** the black ^*^ shows all the plants. Areas of different colors represent the diversity. The areas were generated from the convex hull of the plants belonging to a diversity group.

We perform principal component analysis (PCA) on the EFD. PCA facilitates dimension reduction of the data set and permits efficient summarization of the information contained by the coefficients. The first 30 principal components express around 90% of the total variability, and first and second components describe, respectively, around 17 and 10% of the total variability. These two components are used in the subsequent sections for diversity visualization and interpretation. Figure 7 illustrates the first two PCA coefficients, color-coded according to four different classes (Figure 7A), country of origin; (Figure 7B), maturity index; (Figure 7C), stem termination index; and (Figure 7D), seed weight.

In Figure 7A, lines from China and Japan show the most variation. This is consistent with their status as centers of diversity for the domesticated soybean (Hymowitz and Harlan, 1983; Zhou et al., 2015; Valliyodan et al., 2016). No significant pattern/classification among the countries is observed in the PCs representation in Figure 7A. The reconstructed canopy outlines from 30 PCs, did not show significant diversity among the countries of origin (Figure S1). When grouped in terms of maturity indexes (I, II, or III), our results showed that each maturity group has similar variation of canopy outline, and soybean maturity is not correlated with the canopy outline (Figure 7B). Likewise, similar results were observed for the PCA using SNP genetic marker when country origin and maturity were used to cluster (Figure S2). One possible explanation to this phenomena was that the panel in this study is a subgroup of the USDA soybean core collection and it was designed to maximize the representative of the diversity of all the germplasm lines of maturity index I, II, and III, (Oliveira et al., 2010; Valliyodan et al., 2016). Based on the stem growth habit, the lines are classified into three major categories: determinate (D), semi-determinate (S) and indeterminate (N). For the determinate soybean cultivars, the stem elongation stops soon after photoperiod-induced floral transition of the shoot apical meristem (SAM) from vegetative growth to reproductive growth (Bernard, 1972). In contrast, the transition of SAM to reproductive growth is suppressed in indeterminate cultivars and vegetative growth continues until a cessation is caused by the demand of developing seeds (Tian et al., 2010). Therefore, stem growth habit has broad effects on soybean canopy architecture. Although there is no clear border among subpanels of different stem growth habit, we observed a wide variation of canopy outline in semi-determinate soybean germplasm lines followed by determinate and indeterminate subpanels (Figure 7C). Also, in this study, these images were taken at relatively early vegetative stages of the plants, and differences in canopy architecture related to stem growth habit is less apparent before reproductive stages. Figure 7D indicates that large seeded varieties have significantly less variation in the PC factors, indicating a specific type of ideotype.

### Genetic control of the soybean canopy outline

Genetic improvement of plant canopy has long been a challenge of soybean genetic improvement programs because of the complexity of the trait and the difficulty of measurement. We investigated the genetic effect underlying each of the four shape traits/descriptors. The analysis of variance implied that the genetic effect of all traits, except aspect ratio, were significant (Table 1). Further analysis showed that solidity and roundness have similar, large broad sense heritability, much higher than that of circularity (Table 1). These results suggest that performance of solidity and roundness, rather than aspect ratio and circularity, is predominated by the genetic effects and thus are suitable indexes of soybean canopy improvement. Further dissecting the genetic basis of canopy solidity and roundness will be of great interest.

**Table 1:** **Heritability estimates of the canopy outline descriptors**.

**Trait**	***F_G_*^†^**	**Heritability^‡^**
Aspect ratio	1.10	0.03
Roundness	3.13^***^	0.68
Circularity	1.27^*^	0.22
Solidity	3.46^***^	0.71

† *F_G_ represents the F value for genotypic effects*.

‡ *The estimates are based on 154 lines that have 3 replications*.

* P < 0.05,

*** *P < 0.001*.

## Conclusions

Characterizing and understanding canopy outline variations is important for breeding. This work discusses a framework for efficient representation of complex canopy outlines using EFD. We detail how the choice of the number of harmonics is made to ensure consistency while ensuing a balance with computational effort. We rigorously show that traditional traits/descriptors can be easily, and very accurately reconstructed from the elliptic Fourier representation of the canopy outline. We utilized this framework to explore the diversity in canopy outline using a diverse panel of soybean plants.

In the near term, we envision that this approach will allow for design and utilization of a high throughput canopy morphology phenotyping platform. Such a platform will allow systematic canopy outline analysis while maintaining the integrity of the shape. The use of EFD provides an attractive alternative to conventional canopy phenotypes. Genotypic differences within a species, as well as morphological differences under differing environmental and management conditions, can be characterized without bias, allowing identification of desirable lines/genotypes, as well as providing a proxy for rapidly measuring important plant canopy and growth traits. The approach presented in our paper may also find applications in the hyperspectral and multispectral imaging of disease and other causal damage that is observable on canopy during the crop growing season (Roschera et al., 2016). We also anticipate that this framework will find utility for time series analysis of the canopy throughout the growing season. Our future work is targeted toward providing biological relevance of these canopy traits to soybean productivity and stress tolerance, and thereby their utilization in genetic enhancement. Finally, we think that the approaches presented in paper can be translated to investigations on canopy traits in other crops with similar canopy characteristics to soybean.

## Author contributions

TJ, BG, AKS, and AS formulated research problem and designed approaches. AKS and AS directed field efforts and phenotyping. HN, TJ, JZ, and BG developed processing workflow. BG, AKS, SS, TJ, JS, KP, and JZ performed data analytics. All authors contributed to the writing and development of the manuscript.

## Funding

Partial funding for this work came from Iowa Soybean Association, ISU Plant Science Institute Faculty Fellow (BG), ISU Presidential Initiative for Interdisciplinary Research (PIIR) in Data Driven Science (DDS) project, Monsanto Chair in Soybean Breeding at ISU (AKS), R F Baker Center for Plant Breeding; USDA CRIS project #IOW04314, and the USDA NIFA grant “A multi-scale data assimilation framework for layered sensing and hierarchical control of disease spread in field crops.”

### Conflict of interest statement

The authors declare that the research was conducted in the absence of any commercial or financial relationships that could be construed as a potential conflict of interest. The reviewer ML and handling Editor declared their shared affiliation, and the handling Editor states that the process nevertheless met the standards of a fair and objective review.
