# Supplementary material for: Deploying Fourier Coefficients to Unravel Soybean Canopy Diversity
Source: Front Plant Sci. 2017 Jan 19;7:2066. doi: 10.3389/fpls.2016.02066 (PMC5243820; doi:10.3389/fpls.2016.02066)
Supplement: Supplementary file 1 [file Image1.PDF]

Deploying Fourier coefficients to unravel soybean canopy diversity

Talukder Zaki Jubery[1], Johnathon Shook[2], Kyle Parmley[2], Jiaoping Zhang[2], Hsiang Sing Naik[1], Race Higgins[2], Soumik Sarkar[1], Arti Singh[2], Asheesh K Singh[2]*, Baskar Ganapathysubramanian[1,3,4]*

***Corresponding author: **Baskar Ganapathysubramanian,** baskarg@iastate.edu, and **Asheesh K Singh,** singhak@iastate.edu.

Running title: Soybean canopy shape diversity

.Supplementary Materials

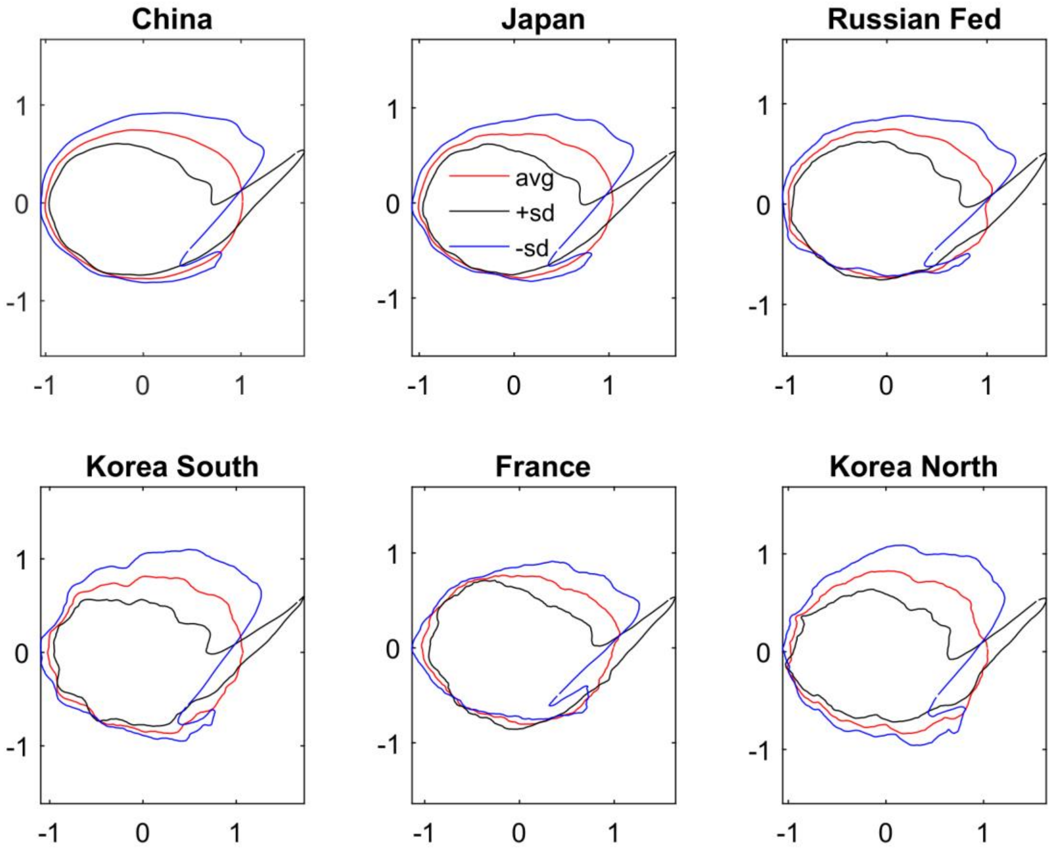

Figure S1 Mean shape of canopy outline and typical variations among the countries. Variation of the reconstructed shapes among plants from different origins. The shape was reconstructed from first 30 principal components of EFD.

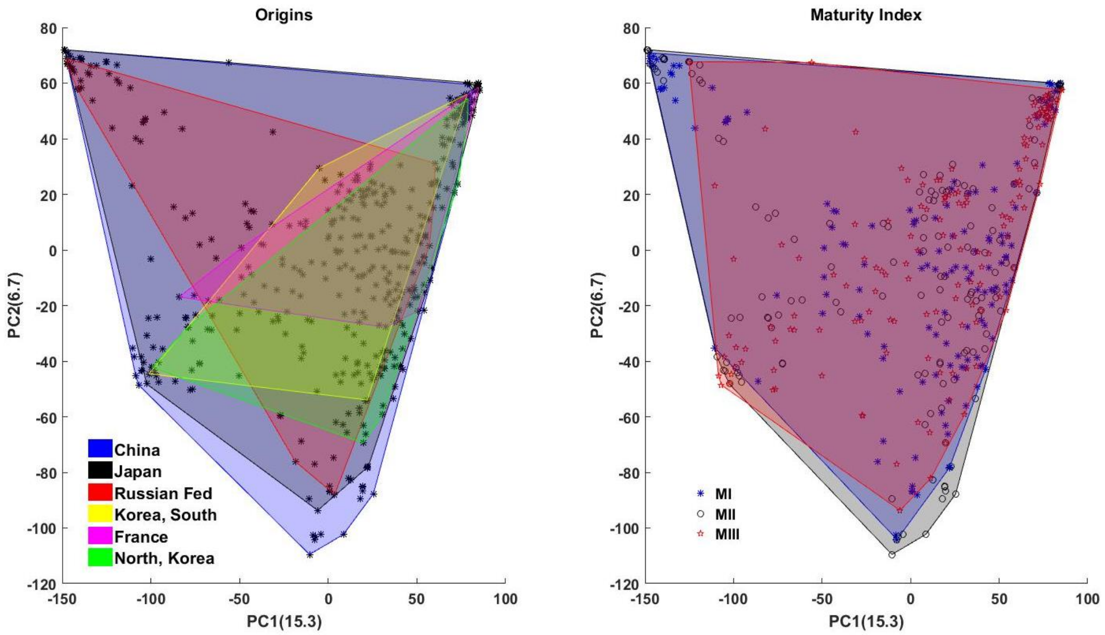

Figure S2 Representation of the diversity of genetic markers among the origins (left) and maturity index (right). Different colors are used to represent different origins or maturity index.
